# Probiotic *Streptococcus thermophilus* FP4 and *Bifidobacterium breve* BR03 Supplementation Attenuates Performance and Range-of-Motion Decrements Following Muscle Damaging Exercise

**DOI:** 10.3390/nu8100642

**Published:** 2016-10-14

**Authors:** Ralf Jäger, Martin Purpura, Jason D. Stone, Stephanie M. Turner, Anthony J. Anzalone, Micah J. Eimerbrink, Marco Pane, Angela Amoruso, David S. Rowlands, Jonathan M. Oliver

**Affiliations:** 1 Increnovo LLC, 2138 E Lafayette Pl, Milwaukee, WI 53202, USA; ralf.jaeger@increnovo.com (R.J.); martin.purpura@increnovo.com (M.P.); 2 Exercise & Sport Performance Laboratory, Department of Kinesiology, Texas Christian University, P.O. Box 297730, Fort Worth, TX 76129, USA; j.stone@tcu.edu (J.D.S.); s.m.turner@tcu.edu (S.M.T.); a.j.anzalone@tcu.edu (A.J.A.); m.eimerbrink@tcu.edu (M.J.E.); 3 R & D Bioloab, Via E. Mattei 3, Novara 28100, Italy; m.pane@mofinalce.it (M.P.); a.amoruso@mofinalce.it (A.A.); 4 School of Sport and Exercise, Massey University, 63 Wallace St., Wellington 6021, New Zealand; d.s.rowlands@massey.ac.nz

**Keywords:** isokinetic, eccentric exercise, inflammation, cytokines

## Abstract

Probiotics have immunomodulatory effects. However, little is known about the potential benefit of probiotics on the inflammation subsequent to strenuous exercise. In a double-blind, randomized, placebo controlled, crossover design separated by a 21-day washout, 15 healthy resistance-trained men ingested an encapsulated probiotic *Streptococcus* (*S.*) *thermophilus* FP4 and *Bifidobacterium* (*B.*) *breve* BR03 at 5 bn live cells (AFU) concentration each, or a placebo, daily for 3 weeks prior to muscle-damaging exercise (ClinicalTrials.gov NCT02520583). Isometric strength, muscle soreness, range of motion and girth, and blood interleukin-6 (IL-6) and creatine kinase (CK) concentrations were measured from pre- to 72 h post-exercise. Statistical analysis was via mixed models and magnitude-based inference to the standardized difference. Probiotic supplementation resulted in an overall decrease in circulating IL-6, which was sustained to 48 h post-exercise. In addition, probiotic supplementation likely enhanced isometric average peak torque production at 24 to 72 h into the recovery period following exercise (probiotic–placebo point effect ±90% CI: 24 h, 11% ± 7%; 48 h, 12% ± 18%; 72 h, 8% ± 8%). Probiotics also likely moderately increased resting arm angle at 24 h (2.4% ± 2.0%) and 48 h (1.9% ± 1.9%) following exercise, but effects on soreness and flexed arm angle and CK were unclear. These data suggest that dietary supplementation with probiotic strains *S. thermophilus* FP4 and *B. breve* BR03 attenuates performance decrements and muscle tension in the days following muscle-damaging exercise.

## 1. Introduction

Probiotics, live micro-organisms, demonstrate modulatory effects on pro- (i.e., IL-6) and anti-inflammatory (i.e., IL-10) cytokines [1,2,3]. Evidence suggests that the immunomodulatory effects are likely strain-specific [4]. The purported anti-inflammatory effects of probiotics have received considerable attention in the athletic community [5]. However, despite that attention, few studies have examined the effect of probiotics in an athletic population. Further, those studies are limited to promoting health over the course of training, only indirectly affecting performance. Jäger et al. [6] recently provided evidence of a potential beneficial effect of probiotics on subsequent performance following a bout of muscle-damaging exercise. 

The training of competitive athletes involves the incorporation of unaccustomed exercise, typically comprising an eccentric component, likely to result in skeletal muscle tissue damage [3,7,8]. Exercise-induced muscle damage occurs as a result of the forced lengthening of active muscle, which directly causes microtears of the myofibrils, thus disrupting the integrity of the sarcolemma [8]. The initial response, known to result in muscle soreness and swelling, and decreased forced production, is followed by a secondary inflammatory response integral to skeletal muscle repair and recovery response [9,10]. While inflammation appears to be an important component of muscular adaptation to exercise [9], athletes under heavy training stress or in tournament situations may benefit from a dampening of the inflammatory response to muscle damage and an accelerated recovery period to support the performance of consequent bouts at maximal intensity. Furthermore, consistent training at competition intensity leads to an enhanced adaptation rate and performance [11]. 

Therefore, the purpose of the present study was to determine the anti-inflammatory properties of co-administration of two probiotic strains (*Bifidobacterium* (*B.*) *breve* BR03 and *Streptococcus* (*S.*) *thermophilus* FP4), which have previously been reported to have anti-inflammatory effects [12,13,14] and have been known to colonize in different areas of the gastrointestinal tract, on measures of skeletal muscle performance, damage, and tension following a bout of strenuous exercise. 

## 2. Materials and Methods 

### 2.1. Subjects

Fifteen (*n* = 15) resistance-trained men (mean ± SD; 25 ± 4 years, 177.9 ± 8.5 cm, 81.1 ± 10.3 kg) completed the study. Inclusion criteria included the following: (1) participation in structured resistance training for 1 year prior; (2) no consumption of any nutritional supplements or ergogenic aids for the preceding 6-week period; and (3) no anti-inflammatory medications for the previous month. This study was conducted according to the Declaration of Helsinki guidelines and registered with ClinicalTrials.gov (NCT02520583). All procedures involving human subjects were approved by the Institutional Review Board of Texas Christian University for use of human subjects in research (protocol No. 1501-009-1502). Written consent was obtained from all subjects. 

### 2.2. Experimental Protocol

A double-blind, randomized, placebo controlled, crossover research design was employed to determine the effect of prior ingestion of probiotic strains *B. breve* BR03 and *S. thermophilus* FP4 on subsequent performance and acute inflammatory response following a bout of muscle-damaging exercise. Prior to experimental testing, a fasting baseline blood sample was taken prior to subjects being randomly assigned to ingest either a probiotic containing 5 bn live cells (AFU) *S. thermophilus* FP4 (DSMZ 18616) and 5 bn live cells (AFU) *B. breve* BR03 (DSMZ 16604) (Probiotical S.p.A., Novara, Italy; Probiotic), based on a prior study dosing regimen [14], or a placebo for 21 days. Study materials were analyzed by Biolab Research S.r.l., Novara, Italy, via flow cytometry (Biolab Research method 612-04, adjusted from ISO 19344:2015 IDF 232:2015 and accredited by Accredia, Rome, Italy, >10 bn live cells), and the plate count method (Biolab Research method 031-08, >10 bn CFU), confirming target cell count. At least 72 h prior to experimental testing, subjects were familiarized with the experimental testing procedures (isometric strength). 

The first experimental trial commenced at least 21 days after initiating supplementation. Subjects were asked to refrain from any activity outside of daily living for the preceding 72 h. Further, subjects were asked to refrain from consuming any nutritional products that may interfere with study procedures. On the day of experimental testing, subjects arrived having fasted (>8 h) and were placed in the supine position for catheter insertion to allow for multiple blood samplings. After a baseline blood sample was obtained, subjects’ upper arm circumference and their range of motion and soreness were assessed prior to performing a warm-up, followed by a determination of isometric strength. Subjects then performed a bout of eccentric exercise of the elbow flexors known to induce muscle damage [15]. The initial limb was chosen at random. Immediately after eccentric exercise, a second blood sample was obtained, and isometric strength was again determined. Subjects were then placed in the supine position for 60 min. Blood samples were obtained at 30 min and 60 min after the bout of eccentric exercise. One hour after exercise, upper arm circumference, range of motion, and soreness were again assessed.

Subjects returned to the laboratory 24, 48, and 72 h after the bout of eccentric exercise for an assessment of blood sampling, upper arm circumference, range of motion, and soreness, followed by a determination of isometric strength. After a 21-day wash-out [16], subjects repeated the experimental protocol with the alternate supplement regimen and the contralateral limb. 

### 2.3. Upper Arm Circumference

In accordance with previously described procedures [17], upper arm circumference was assessed with the subjects’ arm in a relaxed position hanging on the side. A semi-permanent maker was used to mark 3, 5, 7, 9, and 11 cm above the crease line of the elbow of the exercised arm. Two measurements were taken at each point and averaged. The average of all five sites was recorded as the upper arm circumference and used for further analysis. 

### 2.4. Range of Motion

The range of motion of the elbow joint (relaxed arm and flexed arm angle) was obtained with the subjects in a standing position using a goniometer. Relaxed arm angle measurements were taken with the subjects in a standing position, with the exercised arm in a resting position. Subjects were then asked to flex the elbow to touch the shoulder while keeping elbow joint stable at the side to obtain the flexed arm angle. The lateral epicondyle of the humerus, the distal end of the deltoid, the mid-point between styloid process of the ulna and radius, and the styloid process of the radius were used as anatomical landmarks to measure joint angle. In both measurements, the goniometer was applied twice, and the average of the two measurements was used for further analysis [18]. 

### 2.5. Soreness

Ratings of soreness were provided by subjects using a visual analogue scale ranging from 0 (“No Pain”) to 10 (“Worst Possible Pain”). Soreness ratings were provided by subjects at specified time points as described for the exercised and non-exercised arm. 

### 2.6. Isometric Peak

Maximal voluntary isometric peak torque of the elbow flexors was measured at a joint angle of 90° on an isokinetic dynamometer (Biodex System 3; Shirley, NY, USA). Prior to isometric peak torque determination, subjects performed a warm-up of 2 sets of 3 repetitions, 60° to 180°, at two different speeds (90° s^−1^ and 60° s^−1^, respectively) with 30 s of rest between sets. Subjects then performed two tests of isometric peak torque of the elbow flexors, each lasting 4 s separated by 30 s of rest between trials [19]. Verbal encouragement as well as a visual display of force in real time was provided throughout to encourage maximal efforts [20]. 

### 2.7. Eccentric Exercise Protocol

After a warm-up and determination of isometric strength, subjects performed 5 sets of 10 maximal eccentric (forced lengthening) contractions at a speed of 30° s^−1^. After each forced eccentric contraction, from 60° to 180°, research personnel returned the arm to the starting position at a speed of 10° s^−1^ (12 s) so that no concentric contraction was performed by the subject. One minute separated each set, and subjects were encouraged throughout. The same research technician was assigned for all trials involving the same subject. 

### 2.8. Blood Analyses

On the day of the bout of eccentric exercise, subjects were placed in a supine position for catheter insertion. The area was sterilized using standard sterile phlebotomy procedures, and an indwelling catheter (BD Biosciences; San Jose, CA, USA) was inserted into an antecubital vein and capped to allow for multiple blood draws. The catheter was kept patent by flushing with 2–3 mL of 0.9% sodium chloride (G-Biosciences; St. Louis, MO, USA) injected into the portal site. Prior to each blood sampling from the catheter, a 3 mL vacutainer (BD Biosciences; San Jose, CA, USA) was used to withdraw a waste sample. An initial blood sample was obtained prior to initiating supplementation and 21 days later prior to the performance of the bout of eccentric exercise. Thereafter, blood was taken immediately post-exercise and 30 min and 60 min afterwards. Blood samples were also taken at 24, 48, and 72 h post-exercise. Blood samples were drawn into 10 mL EDTA tubes (BD Biosciences, San Jose, CA, USA). Blood samples were centrifuged within 30 min of collection for 20 min at 2000× *g* in a refrigerated centrifuge (4 °C). Aliquots of plasma were then immediately transferred and frozen at −80 °C until further analysis. Plasma samples were assayed for concentrations of IL-6 using a human IL-6 high sensitivity ELISA kit (R & D Systems; Minneapolis, MN, USA). The detection range for this assay was 0.156 pg·mL^−1^ to 10 pg·mL^−1^. The inter- and intra-assay coefficients of variation for each assay were below 10%.

### 2.9. Statistical Analysis

#### 2.9.1. Data Presentation and Transformation

Raw data are presented as mean and standard deviation. All data except the visual analog scale data were log-transformed prior to analyses to manage non-uniformity of error. The effects of treatment and treatment × time on outcomes were estimated from a mixed model analysis of variance (Proc Mixed, SAS 9.4, Cary, NC, USA), with the subject term as the random effect. Estimates of the log-transformed analysis were presented as back log-transformed least-squares means or geometric adjusted means with uncertainty (90% confidence interval, CI). 

#### 2.9.2. Statistical Inference

A magnitude-based approach to inference was utilized [21]. A numerical translation of performance in the current experimental model to performance power is unknown, with isometric torque an investigative model to explore proof of principle. Therefore, the smallest standardized difference was considered the smallest important change for the primary and for the associated mechanistic outcomes. In the present case, the magnitude threshold for the smallest change was the Glass’d standardized difference (0.2 multiplied by the baseline SD for the control condition) [16]. The probability that a contrast was at least greater than the respective thresholds for small was 25%–75% possible, 75%–95% likely, 95%–99.5% very likely, >99.5% almost certain [7,21,22,23]. In the case where the majority (>50%) of the confidence interval (CI) lay between the thresholds for positive and negative substantiveness, the effect was qualified trivial (negligible) [7,21,22,23]. The terms benefit, trivial, and harm refer to the most likely directional outcome, relative to the smallest effect threshold. The term unclear refers to outcomes where the likelihood of both benefit and harm exceeds 5% [22].

## 3. Results

### 3.1. Isometric Peak Torque

**Figure 1:**
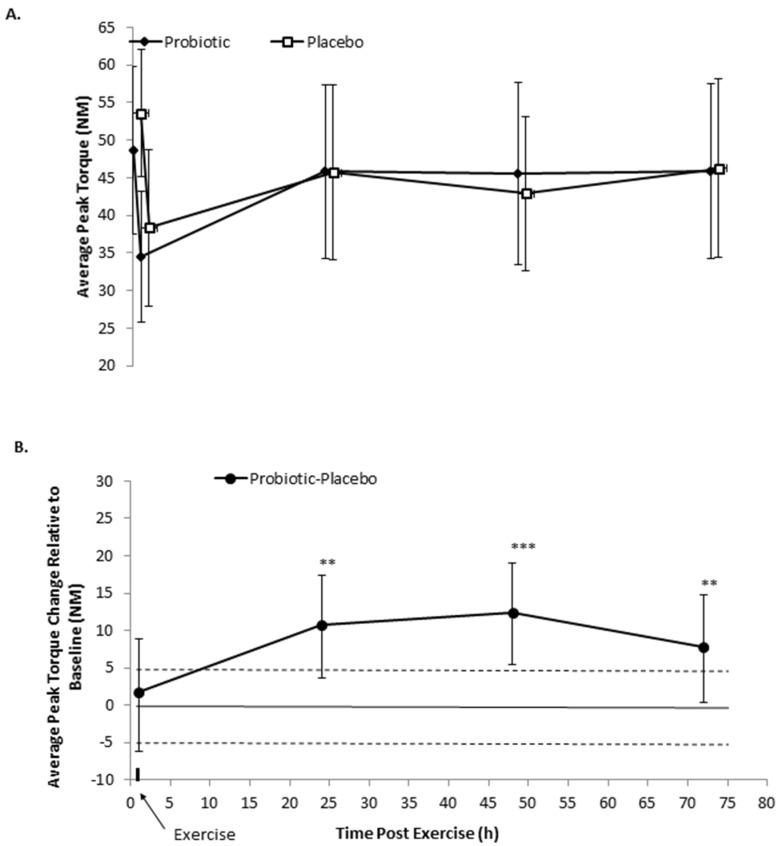
Effect of 21 days of probiotic and placebo supplementation on (**A**) average peak isometric torque before and for 72 h following eccentric loading exercise; (**B**) The probiotic–placebo difference score adjusted for the value at baseline (pre-eccentric exercise). Data in plate A are raw means and SD. Data in plate B are the probiotic–placebo effect expressed as a percent of placebo and 90% confidence intervals derived from the back transformed means from the mixed model analysis of variance. Horizontal dashed lines are the smallest standardized difference threshold (±4.5%), while the probability of substantial change is included above the contrast represented as ** likely, *** very likely; contrasts with no stars are inconclusive or unclear [24].

After 21 days of supplementation, average peak torque was likely lower (13.8%; 90% CI 25.4, 3.3%) following the probiotics relative to the placebo (Figure 1A). Performance decrements were observed following exercise in both conditions, demonstrating the effectiveness of the protocol of inducing damage. Supplementation likely enhanced isometric average peak torque production at 24 to 72 h following damaging exercise, relative to pre-exercise (probiotic–placebo effect and 90% CI, likelihood percent benefit/trivial/harm: 24 h, 11% 90% CI 17, 4%, 92.5/7.4/0; 48 h, 12% 90% CI 19, 55%, 96.5/3.5/0; 72 h, 8% 90% CI 15, 0%, 77.3/22.4/0.3) (Figure 1B). 

### 3.2. Soreness, Range of Motion, and Upper Arm Circumference

**Figure 2:**
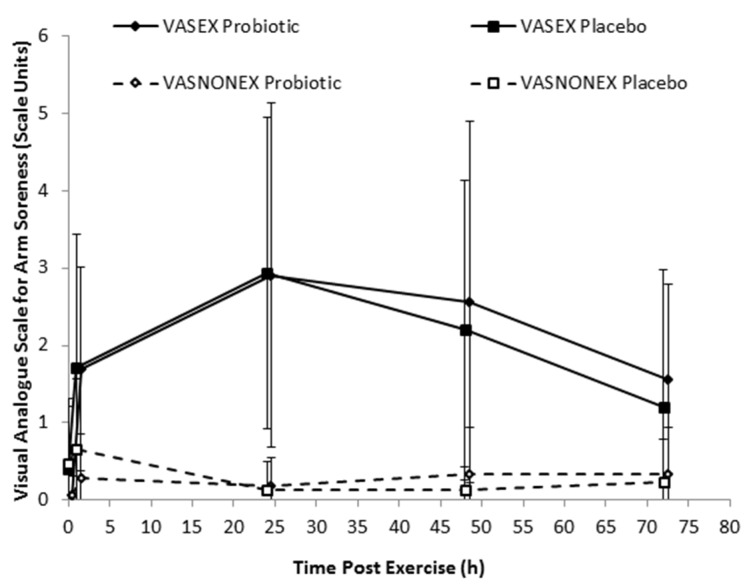
Effect of 21 days of probiotic and placebo supplementation on muscle soreness as measured with the visual analogue scale (VAS) for the exercised (EX) arm and non-exercise (NONEX) arm. Data are raw means and SD. All probiotic–placebo post–pre exercise contrasts were unclear [24].

Exercise almost certainly substantially increased arm muscle soreness from immediate post-exercise to 72 h (average 0.9 to 2.5 scale units; full estimates not shown for brevity), relative to baseline, but the effects of the probiotics at all sample time points were unclear (Figure 2). 

Following exercise, probiotics increased the resting arm angle by a likely moderate effect size at 24 and 48 h, relative to the placebo; meanwhile, effects were unclear for the flexed arm angle (Table 1). 

The effect of probiotic supplementation (pre-exercise time point) on upper arm circumference was likely trivial (full estimates not shown for brevity). The effects on recovery from exercise were also possibly or likely trivial (range of mean probiotic–placebo effect adjusted for pre-exercise score: −0.8%–2.2%; 90% CI range 4.1, −2.9; smallest standardized change 1.5%).

### 3.3. Interleukin-6 and Creatine Kinase 

**Figure 3:**
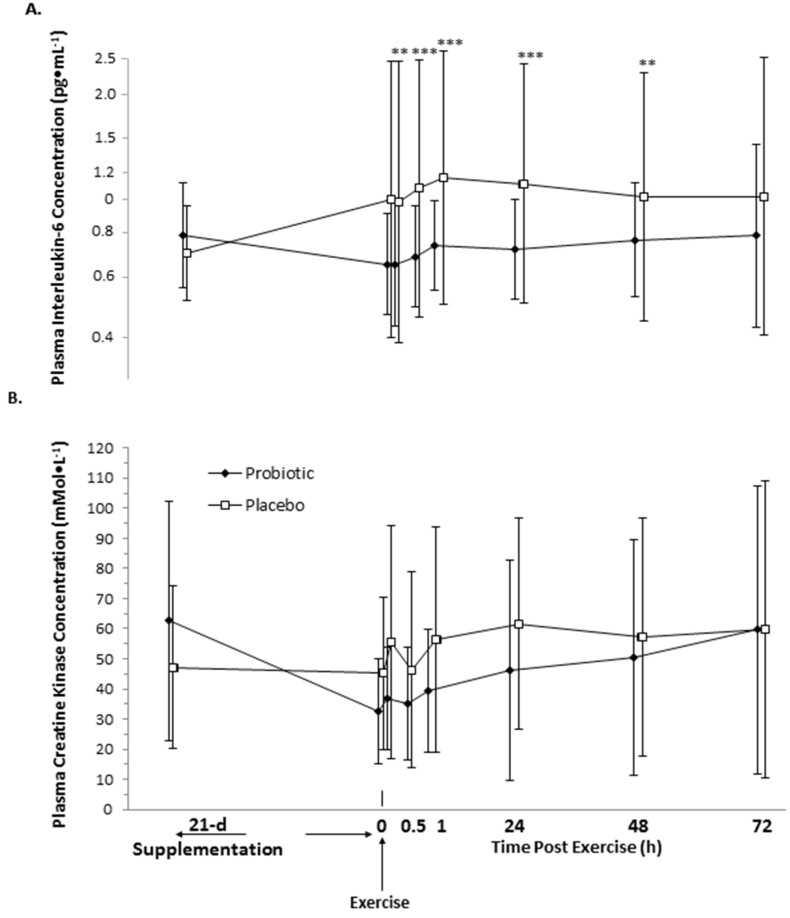
Effect of 21 days of probiotic and placebo supplementation on plasma (**A**) interleukin-6 and (**B**) creatine kinase concentrations. For IL-6, means were generated from log-transformed values and presented on a log scale to manage the heterogeneity of error. Means and SD for creatine kinase concentration are raw. The probability of substantial standardized effect of probiotic–placebo, relative to the baseline is included above the contrast represented as ** likely, *** very likely; contrasts with no stars are inconclusive or unclear. All probiotic–placebo post–pre exercise contrasts were unclear [24].

Relative to baseline, 21 days of probiotic supplementation caused a likely small reduction in IL-6 (−17% 90% CI −33, 2%; likelihoods percent decrease/trivial/increase 79.9/18.8/1.3), whereas IL-6 likely increased in placebo (19% 90% CI −3, 46%; 2.3/17.8/80.0); accordingly, the probiotic–placebo effect was a very likely moderate decrease in IL-6 prior to exercise (−43%, 90% CI −91.3, −7.8%; 95.7/3.6/0.7; threshold for smallest standardized change ±7.8%) (Figure 3A). This effect size on systemic IL-6 relative to baseline was sustained 48 h after exercise (60 min post-exercise: −47% 90% CI −95, −10%, 96.2/2.5/0.5; 24 h −44% 90% CI −92, −8%, 95.9/3.4/0.7; 48 h −25% 90% CI −66, 6%, 80.9/14.4/4.7), but was attenuated to unclear by 72 h (−23%; 90% CI −64, 8%). 

Exercise had no clear effect on IL-6 concentrations relative to the pre-exercise sample, with the exception of a likely increase in IL-6 of 18% (44, 4%; 3.0/20.3/76.7) at 48 h and 21% (1.8/15.3/82.8) at 72 h post-exercise in the probiotic condition; however, there was no clear effect of probiotic–placebo on IL-6 concentrations after exercise (estimates not shown for brevity) (Figure 3A).

21 days of supplementation with probiotics had no clear effect on plasma creatine kinase concentrations prior to eccentric exercise (−9% 90% CI −30, 19%; threshold for smallest standardized change ±16.4%), but there was a likely decrease with the placebo (−45% 90% CI −58, 28%; 99.0/1.0/0) (Figure 3B). Relative to pre-exercise, probiotics increased creatine kinase by a small possible 18% (54, −10%; 2.7/44.4/52.9) immediately post-exercise and 23% (61, −6%; 1.4/34.0/64.5) at 60 min, by a likely 41% (90% CI, 84, 8%; 0.2/10.2/89.7) at 24 h, and a possible 23% (90% CI 61, −8%; 1.5/34.5/64.0) at 48 h and 20% (90% CI 56, −9%; 2.2/49.0/57.0) at 72 h. Creatine kinase concentration also increased in the placebo, relative to pre-exercise, by a likely 29% (90% CI 69, −1%; 0.7/23.4/76.0), 37% (90% CI 80, 4%; 0.3/14.0/85.7), and 51% (90% CI 98, 15%; 0/4.5/95.4) at 24 h, 48 h, and 72 h post-exercise, respectively. However, these differences in the post–pre exercise change scores were not clearly different in the probiotic-placebo contrast (estimates not shown for brevity) (Figure 3B).

## 4. Discussion

The principle finding of the current study was that 21 days of probiotic supplementation with *S. thermophilus* FP4 and *B. breve* BR03 attenuated performance decrements following a bout of muscle-damaging exercise. Although the effect on post-exercise measures of soreness was inconclusive, probiotic supplementation resulted in a greater resting arm angle relative to the placebo. Further, despite a reduction in circulating IL-6 following the 21-day supplementation, probiotics had no additional impact on IL-6 post-exercise. Though post-exercise creatine kinase generally increased, the effect of probiotics was unclear. 

The protocol used in the current study was a modification of those previously reported to result in post-exercise skeletal muscle damage as demonstrated by decrements in performance and a subsequent elevation in creatine kinase, an indirect marker of muscle damage [21]. Our protocol required participants to complete a greater number of forced eccentric repetitions. In agreement with those studies, we observed a decrease in muscle function (quantified as isometric peak torque), increased muscle soreness, and elevations in creatine kinase suggesting damage to skeletal muscle fibers [24,25,26]. Jäger et al. [6] recently reported unclear differences in post-exercise measures of strength following a two-week supplementation with protein in combination with probiotics compared to protein alone. However, those authors did note that power, as measured by the Wingate test, was maintained (+1.7%) post-muscle damage in those supplementing with protein plus probiotics, whereas a decrease (−5.3%) was observed in those ingesting only protein. In agreement, our data show that probiotic supplementation, when not consumed coincident with a protein supplement, attenuates muscle performance decrements, as greater isometric average peak torque was observed relative to the placebo during the several days of recovery following the eccentric lengthening contractions. Accordingly, this is the first study to demonstrate that probiotic supplementation alone attenuates muscle performance decrements.

Heavy eccentric exercise, as used in the current study, is associated with a reduced range of motion and muscle swelling [24]. While changes in circumference—an indicator of muscle swelling—resulted in no clear contrasts, flexed arm angle increased and relaxed arm angle decreased following the exercise bout. Though no clear effect was observed on flexed arm angle with probiotic supplementation, probiotics did increase relaxed arm angle relative to the placebo at 24 and 48 h. A reduced range of motion is an indirect measure of muscle tension (stiffness) and may have a direct impact on performance as it serves as a foundational component of functional movement testing, a commonly implemented tool for predicting injury risks in athletes [25]. Greater scores during functional movement testing and the deep-squat movement positively predicted a percentage change for in-season performance in elite track and field athletes [24]. Those that performed better were purported to possess superior mobility, which, when obstructed, can lead to decreased strength and a decreased range of motion within in a given joint [7,22,23]. Therefore, an increased range of motion, as observed with probiotic supplementation, would therefore be present in subsequent training bouts and may support improved training capacity.

Resting IL-6 concentrations decreased following 21-day probiotic supplementation, while an increase was observed with the placebo. Due to a ubiquitously expressed receptor, IL-6, a pro-inflammatory pleiotropic cytokine, acts on various cells (for a review see [27]). A relationship exists whereby elevated resting concentrations of IL-6 are present in a number of chronic diseased states, such that the IL-6 and IL-6 receptor blockade has emerged as a promising treatment strategy [28]. Thus, the reduction in resting IL-6 observed in the current study may suggest an overall decrease in inflammation. However, the subjects in the current study were all apparently healthy and had baseline IL-6 values within the normal range. Still, the reduction observed herein warrants further study, particularly in those individuals where IL-6 is chronically elevated. In contrast, post-exercise circulating IL-6 increased at 24 and 48 h, but only in the probiotic condition. Though eccentric-based models of muscle damage are recognized, post-exercise changes in inflammatory biomarkers, specifically IL-6, are equivocal [21,29]. This may be due to differences in the exercise, the muscle mass used, or both, as greater post-exercise elevations have been reported with leg extensors [30]. Hirose et al. [31] did not report any significant increases in cytokines, specifically IL-6, post-exercise using a similar eccentric protocol of the elbow flexors. IL-6 is released from exercising skeletal muscle in response to changes in calcium homeostasis, impaired glucose availability, and the formation of reactive oxygen species [32]. Post-resistive exercise elevations of IL-6, a potential regulator of satellite cell function [33], are positively correlated with hypertrophy [28]. The exercise training variables, intensity and more importantly duration, may arguably be the most influential on circulating IL-6 post-exercise [23,34,35]. In the current study, diet was not controlled and subjects reported having fasted, which may have affected the post-exercise response. Therefore, the increase observed at 24 and 48 h may represent an attempt to return to a prior level of circulating IL-6, and further research is warranted in an attempt to elucidate the potential mechanism for these differences. 

In contrast to the observations post-exercise in IL-6, creatine kinase generally increased following the muscle-damaging exercise in both conditions. Creatine kinase concentrations are suggestive of and often utilized as a biomarker for skeletal muscle tissue damage [35]. In a recent study, elevations in creatine kinase were associated with fatigue and strongly correlated with relative work output decrements observed in consecutive sets of muscle-damaging eccentric exercise of the rectus femoris [23]. Hirose et al. [36] reported higher post-exercise concentrations of creatine kinase following a similar eccentric exercise protocol of fewer repetitions. However, subjects in that study were untrained compared with the current study [37]. Further, Chapman et al. [38] reported no post-exercise elevation in creatine kinase using forced lengthening contraction of the biceps femoris to induce muscle damage when velocity was slow (30° s^−1^), but did report substantial increases when the velocity was fast (210° s^−1^), leading those authors to conclude that fast-velocity eccentric exercise causes more muscle damage than slow-velocity exercise in untrained subjects. Jäger et al. [6] reported a smaller increase in creatine kinase post-exercise in those supplementing with protein plus probiotics (138%) compared with those supplementing with protein alone (261%). That study utilized a single-leg exercise bout (such as leg presses, leg extensions, and split squats) for which the eccentric phase would likely have been faster than that used in the current study. Whether a fast eccentric protocol would have induced a similar response and similar attenuation of creatine kinase would have occurred in the presence of probiotic supplementation is unknown.

## 5. Conclusions 

In conclusion, 21 days of probiotic supplementation with probiotics containing 5 bn live cells (AFU) *B. breve* BR03 and 5 bn live cells (AFU) *S. thermophilus* FP4 in healthy, resistance-trained men attenuates performance and the range of motion decrements following an bout of muscle-damaging eccentric exercise. Further, 21 days of probiotic supplementation lowered resting IL-6 concentrations, which was sustained to 48 h post-exercise. These data suggest that the specific dietary probiotics may assist in the recovery of performance following unaccustomed heavy eccentric exercise. Further studies are warranted to elucidate the potential mechanism(s) for these observations and to examine dose response and other exercise conditions.

## Figures and Tables

**Table 1:** Statistical summary for the effect of 21 days of probiotic and placebo supplementation on arm range of motion.

Contrast	Probiotic–Placebo Effect (%) ^a^	90% Confidence Limits (%)		Threshold Smallest Standardized Change	Qualitative Inference ^b^	*p*-Value
		Upper	Lower			
Range of Motion						
Relaxed arm angle						
Pre-exercise	−1.0	0.4	−2.4	0.6	Small decrease likely	0.257
Post–Pre	0.4	2.4	−1.6	0.6	Unclear	0.718
24 h Pre	2.4	4.4	0.4	0.6	Moderate increase likely	0.053
48 h Pre	1.9	3.9	−0.1	0.6	Moderate increase likely	0.116
72 h Pre	0.1	2.1	−2	0.6	Unclear	0.952
Flexed arm angle						
Pre-exercise	2.2	8.3	−4.3	2.1	Unclear	0.571
Post–Pre	−0.3	8.4	−9.9	2.1	Unclear	0.952
24 h Pre	−3.3	5.9	−13.4	2.1	Unclear	0.565
48 h Pre	−1.6	7.5	−11.5	2.1	Unclear	0.783
72 h Pre	−5.6	3.7	−15.8	2.1	Unclear	0.329

^a^ Contrasts are the mean probiotic–placebo effect, with the post-eccentric exercise scores and the post–pre difference score with the 90% confidence interval. Data are the percent effects derived from the back-transformed difference of the log-transformed raw data; ^b^ Magnitude-based inference from Hopkins et al. [21], with the threshold for smallest standardized change shown in the column and the qualified likelihood bins according to the threshold: 25%–75% possible, 75%–95% likely, 95%–99.5% very likely, >99.5% almost certain. Standardized effect size qualifiers were: 0–0.2, small; 0.2–0.6, moderate; 0.6–1.2, large; 1.2–2.0, very large. An unclear effect is logged if the likelihood of both a positive and negative minimally small standardized effect are both >5%.
